# Personalized prediction of the secondary oocytes number after ovarian stimulation: A machine learning model based on clinical and genetic data

**DOI:** 10.1371/journal.pcbi.1011020

**Published:** 2023-04-27

**Authors:** Krystian Zieliński, Sebastian Pukszta, Małgorzata Mickiewicz, Marta Kotlarz, Piotr Wygocki, Marcin Zieleń, Dominika Drzewiecka, Damian Drzyzga, Anna Kloska, Joanna Jakóbkiewicz-Banecka

**Affiliations:** 1 INVICTA Research and Development Center, Sopot, Poland; 2 Department of Biomedical Engineering, Faculty of Electronics, Telecommunications and Informatics, Gdańsk University of Technology, Gdańsk, Poland; 3 Institute of Computer Science, Faculty of Mathematics, Informatics, and Mechanics, University of Warsaw, Warsaw, Poland; 4 MIM Solutions, Research and Development Center, Warsaw, Poland; 5 Department of Medical Biology and Genetics, Faculty of Biology, University of Gdańsk, Gdańsk, Poland; Queen’s University, CANADA

## Abstract

Controlled ovarian stimulation is tailored to the patient based on clinical parameters but estimating the number of retrieved metaphase II (MII) oocytes is a challenge. Here, we have developed a model that takes advantage of the patient’s genetic and clinical characteristics simultaneously for predicting the stimulation outcome. Sequence variants in reproduction-related genes identified by next-generation sequencing were matched to groups of various MII oocyte counts using ranking, correspondence analysis, and self-organizing map methods. The gradient boosting machine technique was used to train models on a clinical dataset of 8,574 or a clinical-genetic dataset of 516 ovarian stimulations. The clinical-genetic model predicted the number of MII oocytes better than that based on clinical data. Anti-Müllerian hormone level and antral follicle count were the two most important predictors while a genetic feature consisting of sequence variants in the *GDF9*, *LHCGR*, *FSHB*, *ESR1*, and *ESR2* genes was the third. The combined contribution of genetic features important for the prediction was over one-third of that revealed for anti-Müllerian hormone. Predictions of our clinical-genetic model accurately matched individuals’ actual outcomes preventing over- or underestimation. The genetic data upgrades the personalized prediction of ovarian stimulation outcomes, thus improving the in vitro fertilization procedure.

## Author summary

Infertility is a condition that leads to the failure of natural conception. It affects more than 186 million people worldwide. Because in vitro fertilization (IVF) is an effective infertility treatment, optimizing the steps in the process is essential to best assist those trying to conceive. The IVF process begins with ovarian stimulation during which the woman takes ovary-stimulating hormones, i.e., gonadotropins, to produce a certain number of viable, fertilization-ready egg cells, but predicting the number of egg cells collected after such stimulation is difficult. These predictions are usually based on the patient’s clinical parameters and depend on the physician’s experience, making them highly subjective. Here, we used machine learning to identify features that physicians could adopt to predict the number of egg cells obtained after ovarian stimulation. We found that clinical parameters (anti-Müllerian hormone level and antral follicle count), as well as genetic characteristics (variants in reproduction-related genes—*GDF9*, *LHCGR*, *FSHB*, *ESR1*, and *ESR2*), are important features that increase the accuracy of such predictions. Our predictive model has been designed to help physicians tailor the protocols used for ovarian stimulation on a case-by-case basis, thereby improving the safety and efficiency of the IVF process.

## Introduction

Infertility affects over 186 million people worldwide and results from a combination of genetic, environmental, and endocrine factors [1]. Clinical parameters used to evaluate the cause of female infertility include endocrine indicators such as follicle-stimulating hormone (FSH), estradiol (E2), inhibin-B, and anti-Müllerian hormone (AMH), as well as ovarian parameters such as ovarian volume, ovarian vascularity indices, or ovarian reserve, and genetic factors, such as karyotype abnormalities or primary ciliary dyskinesia [2–5].

One of the most effective infertility treatments is in vitro fertilization (IVF) and optimization of this technology is essential to support people who cannot conceive naturally. Many male and female factors, including genetic factors, may affect the success of IVF, so reliably predicting its outcome is a challenge. Predictions often depend on the experience of a clinician which makes them highly subjective. Therefore, clinical parameters such as the woman’s age, body mass index (BMI), cause and duration of infertility, and markers of the ovarian reserve such as AMH, FSH, and antral follicle count (AFC) are taken into account in decision-making about IVF treatment [6]. A comprehensive determination of the relationship between these interacting features and the outcome of ovarian stimulation is necessary. Artificial intelligence, machine learning, and deep learning were implemented to develop models used to classify ovarian response [7], select embryos [8], and predict the outcome of embryo implantation or the chances of pregnancy [9]. Fed with patient parameters and IVF cycle-specific variables, predictive models are proving to offer solutions for patients at various stages of the IVF process with significant confidence.

The ovarian response to controlled gonadotropin stimulation and the retrieval of multiple, high-quality oocytes is critical for successful embryo formation, selection, and transfer during the IVF process [10]. The number of oocytes retrieved can increase the probability of obtaining a live birth from an embryo transfer [11]. The dose of gonadotropin and the pool of recruitable follicles are the variables that most affect the number of oocytes collected. A very low dose of gonadotropin is associated with poor ovarian response; raising the dose boosts the number of growing follicles and the oocyte yield, however, very high doses of gonadotropin in women with normal ovarian reserve increase the risk of hyper-response [12]. It seems tempting, therefore, to focus on the woman’s clinical and genetic characteristics to adjust the type or dose of drugs used in ovarian stimulation to obtain an optimal number of oocytes.

The objective of this study was to identify potent predictors of ovarian response to gonadotropin stimulation by assessing the effect of clinical characteristics, cycle-specific parameters, and reproduction-related gene sequence variants on the number of metaphase II (MII) oocytes retrieved. We also aimed to develop and validate a machine-learning model using clinical and genetic data to predict this measure in a cohort of gonadotropin-stimulated patients and oocyte donors.

## Materials and methods

### Ethics statement

The study was conducted according to the guidelines of the Declaration of Helsinki and approved by the Ethics Committee of the Regional Medical Chamber of Gdańsk (protocol code KB-23/20, date of approval 27^th^ October 2020). Written informed consent was obtained from all individuals involved in the study. All individual-level data, including clinical data, were de-identified.

### Study population

Data for the study was collected between November 2014 and February 2021 at INVICTA Fertility Clinics (Bydgoszcz, Gdańsk, Gdynia, Słupsk, Warszawa, Wrocław; Poland). The study population consisted of 6,043 women (9,090 IVF processes) diagnosed with infertility undergoing controlled ovarian stimulation with menotropin (Menopur; Ferring GmbH, Kiel, Germany), follitropin delta (Rekovelle; Ferring GmbH, Kiel, Germany), or follitropin alfa (Gonal F; Merck Serono S.p.A., Modugno, Italy). Exclusion criteria were: stimulation protocols with other gonadotropins, AMH levels above 15 ng/ml, or undetected.

### DNA extraction

Genomic DNA was isolated from whole blood or urogenital swabs using the MagNA Pure 96 IVD instrument and the MagNA Pure 96 DNA and Viral NA Small Volume Kit (Roche, Basel, Switzerland). DNA concentration was quantified with the Qubit 2.0 fluorometer (Life Technologies, Rockville, MD, USA) and Qubit dsDNA HS Assay Kit (ThermoFisher Scientific, Waltham, MA, USA). Isolates were stored at −80°C.

### Genotyping

Sequence variants were identified by next-generation sequencing (NGS). Targeted libraries were prepared from 10 ng of gDNA using the Ion AmpliSeq Library Kit 2.0 (ThermoFisher Scientific, Waltham, MA, USA) and Ion AmpliSeq Made-to-Order panel (primer set version 7.05 based on the hg38 reference genome including 325 amplicons in three primer pools, covering approximately 73,000 bases in 121 exons with 100-bp exon padding of 14 genes of interest). Sequencing was conducted using the Ion Personal Genome Machine (PGM) (Life Technologies, Carlsbad, CA, USA) and Ion PGM Hi-Q View Sequencing Kit (ThermoFisher Scientific, Waltham, MA, USA). Data were analyzed with Ion Torrent Suite Server software version 5.12.2 (ThermoFisher Scientific, Waltham, MA, USA). Technical details are provided in **S1 Appendix**.

### Machine learning for a predictive model

A machine learning model was based on the gradient boosting machine (GBM) technique [13] implemented by the LightGBM framework [14]. The GBM has a built-in ability to handle missing data and in this study, data from the previous stimulation were not available for the first-time-stimulated patients. To keep all observations in the modeling, we decided to use GBM. As GBM parameters, 100 decision trees with five leaves and a maximum depth of 16 were selected. The training was performed with *l*_2_ loss function and gradient-boosted decision trees (GBDT) using the k-fold cross-validation method with five folds.

Error metrics used to validate model performance included the root mean square error (RMSE), the mean absolute error (MAE), and the mean absolute percentage error (MAPE) (defined in **S2 Appendix**). The SHapley Additive exPlanations (SHAP) library [15] was used to explain model predictions.

### Statistical tests

The Kolmogorov–Smirnov and the Mann–Whitney U tests were used to determine differences in the distribution of the number of MII oocytes among patients carrying the reference or alternative allele of sequence variants (hypotheses defined in **S2 Appendix**). The Pearson correlation coefficient (*r*), along with the two-sided *p*-value, was determined to investigate the linear association between clinical variables and the number of MII oocytes. The significance level was set to α = 0.05.

### Ranking methods

Boruta [16] with the random forests (RF) algorithm [17] and Boruta-SHAP [18] with the GBM algorithm were used to rank sequence variants. Each sequence variant was considered a separate feature with the alternative allele denoted as 1; the number of MII oocytes was considered a target feature. A rating was assigned to variants according to their importance in predicting the number of MII oocytes.

### Correspondence analysis

Correspondence analysis (CA) [19] was used to find sequence variants characteristic of patient groups. Inertia covered I ∈ [0,1] was used to assess the goodness of the model’s representation of the dataset, where 1 is a perfect representation. The identified genetic feature was defined as:

$$IV-CA=\sum_{variant\in selected\;variants}\{\begin{array}{c} 1\;if\;variant\;is\;alternative\\ 0\;if\;variant\;is\;reference \end{array}$$ (1)

### Generation of a self-organizing map

A self-organizing map (SOM) [20] was applied to detect the most frequent sequence variants in patient groups. The model was trained with the following parameters: size 6×6 neurons, hexagonal topology, Gaussian neighboring function, sigma = 1.5, lr = 0.7, 100,000 train iterations, and Manhattan distance metric. The best matching unit (BMU), defined as a neuron with the lowest distance to observation was assigned for each IVF process. The quantization error (QE) metric (defined in **S2 Appendix**) was used to validate SOM performance. The identified genetic feature was defined as:

$$SOM_{v}=\sum_{variant\in selected\;variants}\{\begin{array}{c} 1\;if\;variant\;is\;alternative\\ 0\;if\;variant\;is\;reference \end{array}$$ (2)

A standardized version of the feature was used for training:

$$IV8-6=\frac{SOM_{v}-E(SOM_{v})}{\sigma (SOM_{v})}$$ (3)

### Haplotype construction

Each chromosome was analyzed separately using Haploview software [21] with confidence intervals, the four gamete rule, and solid spine of linkage disequilibrium (LD) as block generation algorithms. Uniform Manifold Approximation and Projection (UMAP) [22] was used to reduce the number of variants in haplotypes. The K-means algorithm was used to cluster the observations and separate groups of similar patients. Observations were classified based on the genetic data using a decision tree [23]. Variants in the decision nodes between the root node and leaves created reduced haplotype defined as:

$$reduced\;haplotype=\left\{ \begin{array}{c} 1\;if\;all\;variants\;correspond\;to\;baseline\;haplotype\\ 0 \end{array}\right.$$ (4)

## Results

### A preliminary predictive model based on clinical data

**Fig 1:**
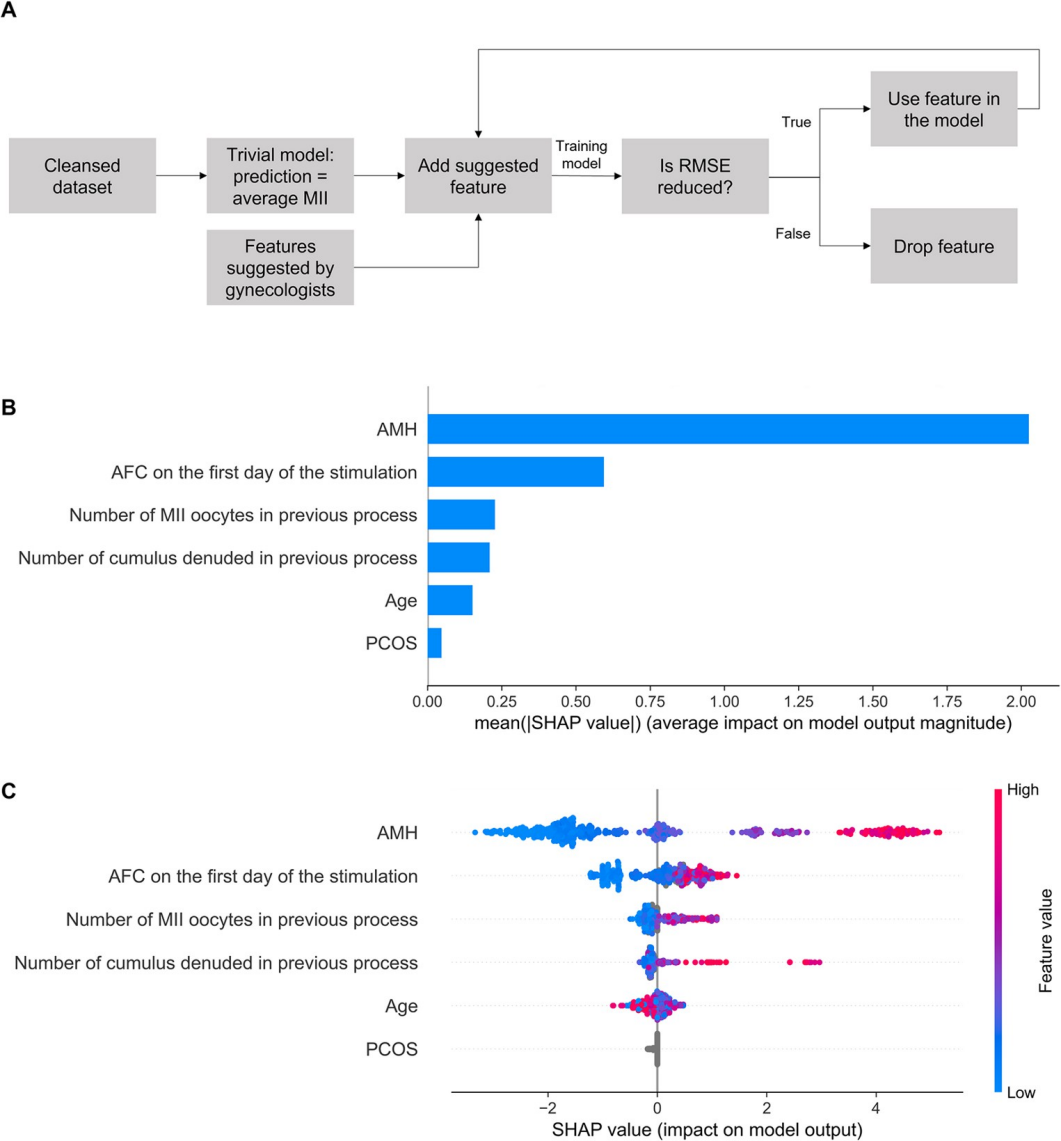
Clinical model for prediction of the number of MII oocytes. **(A)** Feature selection of clinical features. **(B)** The importance ranking of the top six features according to the mean absolute SHapley Additive exPlanations (SHAP) value (|SHAP value|). **(C)** The effect of features on the outcome of the model. The higher the SHAP value of a feature, the higher the number of MII oocytes. A feature takes values from low (blue) to high (red). The feature ranking (y-axis) indicates their importance in the predictive model. The SHAP value (x-axis) is a unified index that responds to the influence of a certain feature in the model. For each feature, the attributions of all patients to the outcome are drawn with dots, where red represents the high-risk value and blue represents the low-risk value. AFC—antral follicle count; AMH—anti-Müllerian hormone; PCOS—polycystic ovary syndrome; RMSE—root mean squared error.

The study population was divided into two groups according to the availability of genetic data: Group 1, without genetic data, included 5,779 patients and 8,574 IVF processes; Group 2, with genetic data, included 264 patients and 516 IVF processes. All women were between 18 and 46 years old, had regular 26–32-day menstrual cycles, were undergoing their first or second IVF cycle, and exhibited no signs of androgenicity, endometriosis, or any chronic diseases. Baseline clinical characteristics are presented in **Table 1**. Clinical data, hormonal test results, data on previous IVF processes and basic characteristics of women in Group 1 (**S1 Table**) were used to construct the preliminary model to determine the most important features for predicting the number of MII oocytes retrieved after ovarian stimulation. The RMSE metric calculated for the trivial model, defined as the expected number of MII oocytes equal to the population average, was 4.75 oocytes (the model predicts the number of MII oocytes ± 2.375 oocytes); it was considered a benchmark value. Features were then added to the dataset in the iterative process and a model was trained in each iteration. If the model’s RMSE was reduced by adding a new feature, the feature was considered important for the prediction (**Fig 1A**). The final set of clinical features selected using this approach consisted of AMH, AFC on the first day of stimulation (follicles ≤10 mm), age, number of MII oocytes in the previous pick-up, number of cumulus-denuded oocytes in the previous pick-up, and polycystic ovary syndrome (PCOS).

**Table 1:** Baseline clinical characteristics of study groups. AMH—anti-Müllerian hormone, BMI—body mass index, E2—estradiol, FSH—follicle-stimulating hormone, IU—international unit, IVF—in vitro fertilization, LH—luteinizing hormone.

Characteristic	Group 1 without genetic data Mean ± SD	Group 2 with genetic data Mean ± SD
No. of women	5,779	264
No. of IVF processes	8,574	516
Woman’s age (years)	34.51 ± 4.54	34.55 ± 4.20
BMI (kg/m^2^)	23.42 ± 4.62	23.15 ± 4.11
Stimulation days	8.79 ± 4.36	9.77 ± 3.10
FSH (mIU/mL)	6.46 ± 2.83	7.25 ± 3.53
LH (mIU/mL)	6.23 ± 4.63	6.10 ± 4.63
E2 (pg/mL)	18.62 ± 18.50	16.62 ± 16.73
AMH (ng/mL)^a^	2.98 ± 2.42	3.38 ± 3.67
No. of follicles (≤ 10 mm) on the first day of stimulation	14.63 ± 8.99	13.41 ± 10.24
No. of cumulus-denuded oocytes retrieved in previous stimulation	8.26 ± 5.26	7.55 ± 6.15
No. of MII oocytes retrieved in previous stimulation	6.17 ± 4.10	5.85 ± 4.59
No. of cumulus-denuded oocytes retrieved	9.14 ± 5.82	7.67 ± 6.08
No. of MII oocytes retrieved	6.96 ± 4.64	5.92 ± 4.75
Cumulative gonadotropin dose (IU)	2,109.76 ± 826.62	2,347.36 ± 795.42

a) observations with an AMH level above 15ng/mL were removed from the database.

Based on these features, two versions of a machine learning model predicting the number of MII oocytes were trained. The first model was trained on the Group 1 dataset and achieved the precision metrics RMSE = 3.80, MAE = 2.85, and MAPE = 0.65. The second model was trained on Group 2 dataset using only selected clinical features (the model is referred to as the clinical model). To assess the impact of genetic data, we compared error metrics for models trained on the same group of patients (Group 2 without genetic data vs. Group 2 with genetic data).

The clinical model achieved better precision metrics (RMSE = 3.53, MAE = 2.58, and MAPE = 0.71) compared to the benchmark value. Feature importance ranking and the effect on prediction for the clinical model are presented in **Fig 1B** and **1C**. The AMH is the most important feature for the prediction and its higher levels result in a higher number of MII oocytes, although the variance is still high. The second most important predictor is the AFC on the first day of stimulation. The third, although much less important feature, is the number of MII oocytes retrieved in the previous stimulation. Although the patient’s age is a less important feature, we observed that the AMH level decreases with age (*r* = −0.17, *p* < 0.01) but the rate differs between patients. The number of MII oocytes correlates with AMH (*r* = 0.64, *p* < 0.01) better than with age (*r* = −0.18, *p* < 0.01), and therefore the model prefers to follow correlations with AMH.

### Sequence variants important for predicting the number of MII oocytes

To improve the prediction, we decided to include data on sequence variants in reproduction-related genes in our analysis. Fourteen genes were selected such as *AMH*, *AMHR2*, *AR*, *BMP15*, *ESR1*, *ESR2*, *FSHB*, *FSHR*, *GDF9*, *LHB*, *LHCGR*, *PRL*, *PRLR*, and *SOX3*, annotating to the Gene Ontology (GO) categories such as reproductive structure development, hormone-mediated signaling pathway, ovulation cycle process, signaling receptor binding or signaling receptor regulator activity (**S2 Table**).

**Fig 2:**
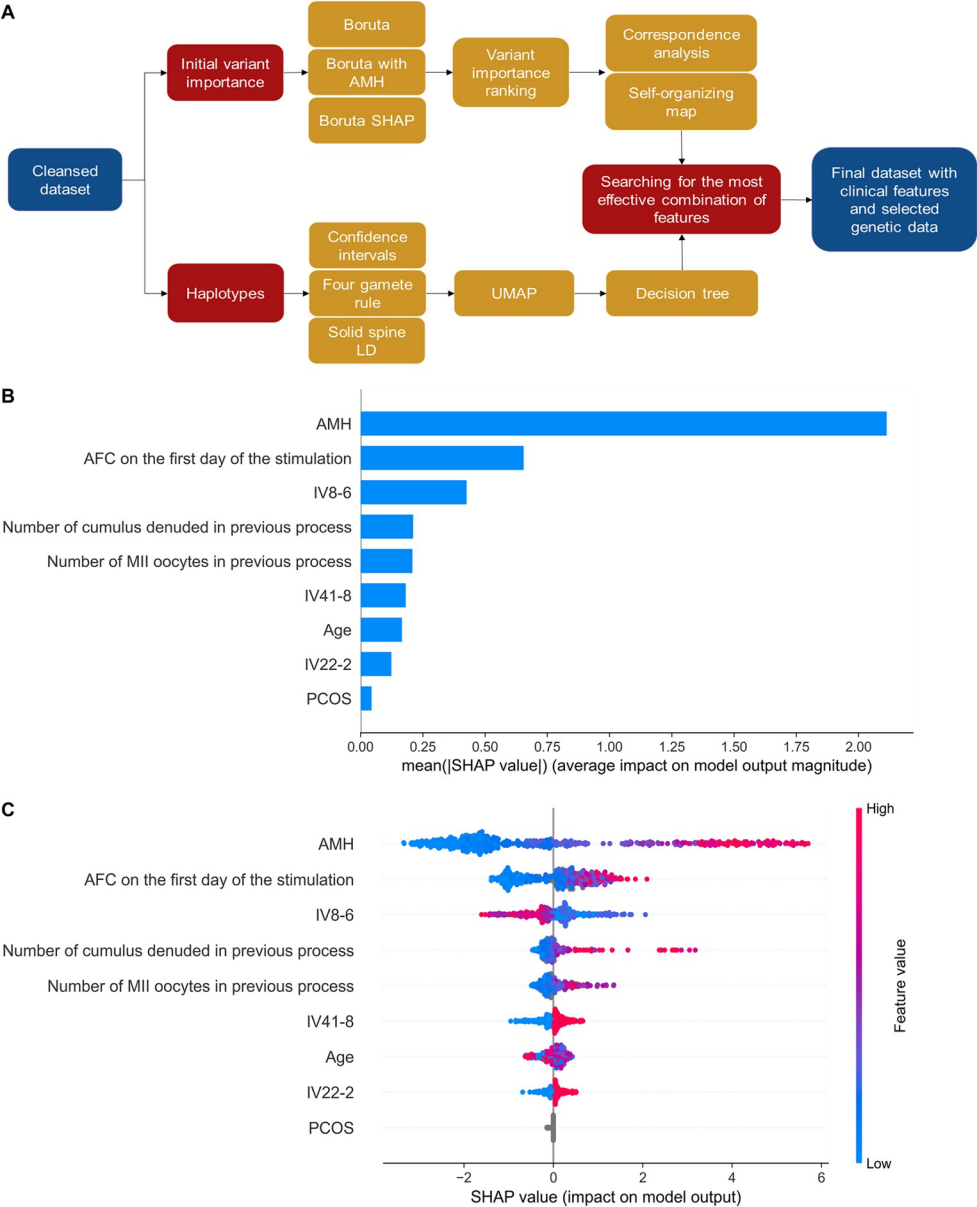
Clinical-genetic model for prediction of the number of MII oocytes. The model was trained using clinical and genetic features (IV8-6, IV41-8, and IV22-2) identified as important for the prediction. (A) The overall flowchart for the approach used to generate sequence variant combinations for implementation in the predictive model. (B) The importance ranking of features according to the mean SHapley Additive exPlanations (SHAP) value (|SHAP value|). (C) Interpretation and stability of important features using the optimal model. The higher the SHAP value of a feature, the higher the number of MII oocytes. The red part of the feature value represents a higher value. The feature ranking (y-axis) indicates the importance of the predictive model. The SHAP value (x-axis) is a unified index that responds to the influence of a specific feature in the model. In each row, the attributions of all patients to the outcome are drawn with dots of different colors, where the red dots represent the high-risk value and the blue dots represent the low-risk value. AFC—antral follicle count; AMH—anti-Müllerian hormone; LD—linkage disequilibrium; PCOS—polycystic ovary syndrome; SHAP—SHapley Additive exPlanations; UMAP—Uniform Manifold Approximation and Projection.

A total of 544 sequence variants were identified in Group 2 by genotyping (**S3 Table**). Our approach to finding variants important for predicting the number of MII oocytes consisted of pre-selection with statistical tests or ranking methods, and identification of haplotypes or variant combinations important for the prediction followed by checking the performance of a model trained with clinical and genetic data (**Fig 2A**).

Kolmogorov–Smirnov and Mann–Whitney U tests revealed eight sequence variants that result in a statistically higher number of MII oocytes and 18 sequence variants that result in a statistically lower number of MII oocytes (**S4** and **S5 Tables**). However, when individually included in the modeling dataset, these variants showed little or no improvement in the model’s error metrics. Next, the pre-selection of variants important for the prediction was performed with Boruta and Boruta-SHAP ranking methods on a dataset of all variants; the number of MII oocytes was a target feature. The Boruta was also applied to a dataset of variants and AMH levels. Variants were ranked according to their importance for the prediction with the final ranking created by re-sorting the combined score. The top 20 variants were selected for further analysis (**S6 Table**).

### Combinations of sequence variants important for predicting the number of MII oocytes

The sequence variants selected by ranking methods were used to search for combinations of variants associated with high numbers of MII oocytes. According to the number of MII oocytes, IVF processes were categorized as MII <2, (2,4], (4,7], (7,11], and 11< (groups contained 144, 101, 122, 85, and 64 IVF processes, respectively). Correspondence analysis and self-organizing map algorithms were used to determine the combinations characteristic of patients with the lowest and highest oocyte numbers (<2 and >11 MII groups).

In correspondence analysis, the more the sequence variant is characteristic of a given MII group, the closer to the group it is located in the graph (**S1 Fig**). Based on this analysis, we defined a new genetic feature, referred to as IV-CA, consisting of three alternative variants in *LHCGR* rs11887058, *PRLR* rs112461, and *ESR1* rs2207396, located closest to the group with above 11 MII oocytes retrieved (**Table 2**).

**Table 2:** Genetic features identified as important for the prediction of the number of MII oocytes after ovarian stimulation.

Genetic feature	Method^*a*^	Gene	dbSNP ID^*b*^	Sequence variant	Allele frequency^*c*^
IV-CA	CA	*LHCGR*	rs11887058	NC_000002.12:g.48729336C>T	T = 0.428
		*PRLR*	rs112461	NC_000005.10:g.35063190A>T	T = 0.242
		*ESR1*	rs2207396	NC_000006.12:g.152061247G>A	A = 0.356
IV8-6	SOM	*GDF9*	rs11739194	NC_000005.10:g.132865538T>C	C = 0.515
			rs17166294	NC_000005.10:g.132866205T>C	C = 0.288
		*LHCGR*	rs11887058	NC_000002.12:g.48729336C>T	T = 0.428
		*FSHB*	rs676349	NC_000011.10:g.30234435A>G	G = 0.523
		*ESR1*	rs2273207	NC_000006.12:g.152061190A>G	G = 0.117
		*ESR2*	rs928554	NC_000014.9:g.64227477C>T	T = 0.731
IV22-2	Haplotype	*FSHR*	rs80111020	NC_000002.12:g.48962060 =	A = 0.909
			rs6166	NC_000002.12:g.48962782C>T	T = 0.701
IV41-8	Haplotype	*PRLR*	rs387032	NC_000005.10:g.35061629 =	T = 0.845
			rs401694	NC_000005.10:g.35062516 =	C = 0.765
			rs112461	NC_000005.10:g.35063190 =	A = 0.758
			rs1057828	NC_000005.10:g.35064413 =	C = 0.939
			rs56251626	NC_000005.10:g.35064922 =	C = 0.936
			rs62355478	NC_000005.10:g.35065548 =	C = 0.992
			rs78373811	NC_000005.10:g.35068146 =	G = 0.913
			rs186609463	NC_000005.10:g.35069864 =	G = 0.985
IV16-3	Haplotype	*GDF9*	rs75061517	NC_000005.10:g.132866082 =	T = 0.689
			rs17166294	NC_000005.10:g.132866205 =	T = 0.712
			rs30177	NC_000005.10:g.132866719C>G	G = 0.924

*a*—features were determined using correspondence analysis (CA), self-organizing map (SOM) algorithm, and haplotype analysis followed by a reduction of the number of variants

*b*—dbSNP Reference SNP number according to the National Center for Biotechnology Information (NCBI) dbSNP database (https://www.ncbi.nlm.nih.gov/snp/, accessed: 2022-04-05)

*c*—allele frequencies were calculated for Group 2 consisting of 264 women.

The SOM algorithm detected the most frequent sequence variants in neurons, where the given MII group was over-represented (**S2 Fig**). The 10 most characteristic variants, with the biggest percentage difference between the frequency of alternative variants in a node and the percentage share in the entire population, were found for *GDF9* rs11739194, *ESR2* rs928554, *ESR1* rs2077647, *GDF9* rs17166294, *FSHB* rs676349, *ESR1* rs2207396, *LHCGR* rs62137532, *ESR1* rs2273206, *LHCGR* rs11887058, and *ESR1* rs2273207. Of all variant combinations tested in terms of the effect on the predictive model, a set of six variants reduced the model’s RMSE metric and comprised one genetic feature important for predicting the number of MII oocytes, further referred to as IV8-6 (**Table 2**).

The IV-CA and IV8-6 features are non-binary: feature values for modeling are determined from the number of alternative alleles among the variants comprising the feature.

### Haplotypes important for predicting the number of MII oocytes

We decided to test whether the sequence variants identified by genotyping could be arranged into haplotypes relevant for predicting the number of MII oocytes. Each of the 311 potential haplotypes generated in Haploview software was analyzed for the effect on the RMSE metric of the predictive model. Three haplotypes, generated by the 4 Gamete Rule and consisting of variants in the *FSHR*, *PRLR*, and *GDF9* genes (**S7 Table**) showed the greatest impact on the prediction. Identified haplotypes increase the number of MII oocytes retrieved.

Haplotypes were composed of a relatively large number of variants, thus, we used the UMAP grouping algorithm and a decision tree to identify a subset of variants in each haplotype, which is sufficient to distinguish patient groups (**S3 Fig**). Reduced haplotypes, referred to as genetic features IV22-2, IV41-8, and IV16-3 (**Table 2**) occurred in the study population with a frequency of 0.58, 0.65, and 0.17, respectively. Haplotypes are considered binary features: if any detected variant is different from the variants comprising the haplotype, the feature value for modeling is set to 0.

### A predictive model based on clinical data and genetic features

To validate the contribution of genetic features in predicting the number of MII oocytes, a total of 127 combinations of genetic features IV-CA, IV8-6, IV22-2, IV41-8, and IV16-3 were used in addition to clinical data in an iterative process. The addition of any genetic feature decreased the model’s RMSE metrics compared to the clinical model (**S8 Table**). The best combination of genetic features included the SOM-identified IV8-6 feature and haplotypes IV41-8 and IV22-2, for which the RMSE metric was reduced by 0.18 oocytes, which corresponds to 5% of the error metric.

The final clinical-genetic predictive model, trained on the dataset of selected clinical and genetic features, achieved lower error metrics (RMSE = 3.35, MAE = 2.48, and MAPE = 0.68) compared to the clinical model. Still, the AMH was the first, and AFC on the first day of stimulation was the second most important feature for the prediction (**Fig 2B**). The combined contribution of all three genetic features appeared to be over one-third of that revealed for AMH. The IV8-6 feature ranked third in terms of importance with the impact on prediction close to that of AFC. The two haplotypes had a lower impact on the prediction (**Fig 2B**), but each haplotype increased the number of MII oocytes (**Fig 2C**). Interestingly, we found a correlation between the genetic feature IV8-6 and the AMH level; with an increased AMH, the effect of the feature on the number of MII oocytes is more apparent (**S4 Fig**). The highest values in the IV8-6 feature decrease the predicted number of MII oocytes while feature values around 0 tend to increase the predictions (especially for patients with high AMH). The lowest IV8-6 values do not affect the prediction.

### Interpretation of personalized predictions

**Fig 3:**
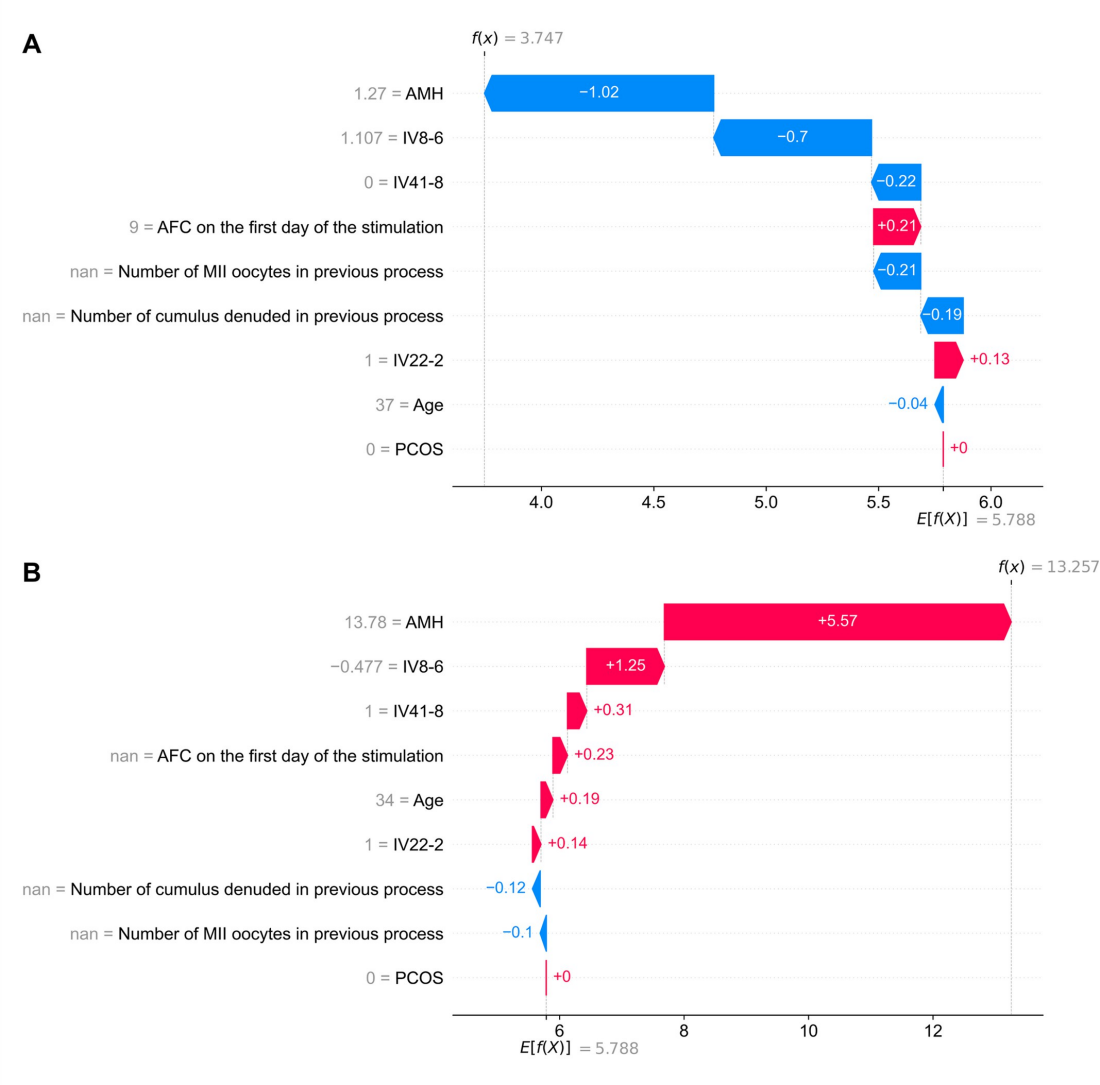
Interpretation of two examples of personalized predictions of the number of MII oocytes. The effect of all features used in the clinical-genetic predictive model on the predictions of the number of MII oocytes was estimated retrospectively for (**A**) Patient 1 and (**B**) Patient 2. The x-axis shows the number of MII oocytes, and the y-axis shows the included features. The arrows indicate the contribution of each feature to the final prediction *f*(*x*) obtained for a patient; red and blue indicate an increase or decrease in the predicted number of MII oocytes by the factor, respectively. A detailed interpretation of both cases is provided in the text. AFC—antral follicle count; AMH—anti-Müllerian hormone; nan—not a number; PCOS—polycystic ovary syndrome.

Patient 1 is a 37-year-old woman with AMH = 1.27 ng/ml, AFC (below 10 mm) on the first day of the stimulation = 9, with no PCOS and no data on previous stimulation results. Haplotype IV22-2 was present while haplotype IV41-8 was absent. Four sequence variants had alternative alleles for the IV8-6 feature—*GDF9* rs11739194 (T>C), *LHCGR* rs11887058 (C>T), *FSHB* rs676349 (A>G), *ESR2* rs928554 (C>T). The patient grew three MII oocytes after stimulation while our clinical-genetic model predicted 3.75 MII oocytes (**Fig 3A**). A relatively low AMH lowered the prediction by 1.27. The next two most impactful features were IV8-6 and IV41-8, which lowered the prediction by 0.7 and 0.22, respectively. The AFC and the haplotype IV22-2 increased the prediction by 0.21 and 0.13, respectively. Age had a very low effect compared to other features. The prediction was higher by 0.75 compared to the actual number of MII oocytes retrieved. The genetic data had a cumulative impact of −0.79, therefore, the prediction would be twice as inaccurate without this information.

Patient 2 is a 34-year-old woman with AMH = 13.78 ng/ml, with no PCOS, and no data on AFC (below 10 mm) on the first day of the stimulation nor previous stimulation results. Haplotypes IV22-2 and IV41-8 were present. Two of the sequence variants had alternative alleles for feature IV8-6—*LHCGR* rs11887058 (C>T) and *ESR2* rs928554 (C>T). The patient grew 13 MII oocytes after stimulation while our clinical-genetic model predicted 13.26 MII oocytes (**Fig 3B**). A relatively high AMH increased the prediction by 5.57. The next two most impactful features were IV8-6 and IV41-8, which increased the prediction by 1.25 and 0.31, respectively. Age and haplotype IV22-2 increased the prediction by 0.19 and 0.14, respectively. The prediction was higher by 0.26 than the actual number of MII oocytes retrieved. Genetic data had a cumulative impact of 1.7, therefore, the prediction would be an underestimation without this information.

## Discussion

Here, we identified sequence variants with a significant impact on the number of MII oocytes retrieved after ovarian stimulation and improved the precision of a model for predicting this measure by including data on genetic variation in reproduction-related genes.

The risk of poor or excessive ovarian response after controlled stimulation remains a serious problem in reproductive medicine. Although the poor response (< 4 oocytes retrieved) occurs in 5 to 35% of women [24], higher doses of gonadotropins to retrieve more oocytes can lead to ovarian hyperstimulation syndrome [25]. Predictors providing a more accurate estimate of a woman’s ovarian response are being sought to optimize individual stimulation protocol before a given IVF cycle [10]. Since the low number of MII oocytes reduces the rates of fertilization, embryo development, pregnancy, and live birth [26], we chose this parameter as an outcome measure of ovarian stimulation.

AFC and AMH are used to predict ovarian response to gonadotropin stimulation most often [6]. Both correlate strongly with each other and the number of oocytes retrieved [27] but a discordance between AFC and AMH is observed in one in five patients [28] making the prediction less accurate. The woman’s age, levels of FSH and AMH, AFC, gonadotropin dose, or type of stimulation protocol increase the accuracy of personalized predictions [29]. According to our study, the number of MII oocytes in the previous pick-up, and the number of cumulus-denuded oocytes in the previous pick-up are also strong predictors of the ovarian stimulation outcome. Combining these features in a single model ensures its high accuracy. Although studies have suggested that PCOS also affects the number of retrieved oocytes [30], our study did not identify this condition as relevant for prediction. This discrepancy may be attributed to the low percentage of patients diagnosed with PCOS in our study population. PCOS also tends to correlate more strongly with the number of total oocytes than the mature MII oocytes retrieved [30] and here we chose the latter as the target feature.

Genetic variation makes a noteworthy contribution to ovarian response. Single nucleotide polymorphisms have been identified in genes with key roles in oogenesis, folliculogenesis, and female reproduction, such as in estrogen receptor 2 (*ESR2*), follicle-stimulating hormone receptor (*FSHR*), FSH β-chain (*FSHB*), luteinizing hormone β-chain (*LHB*), LH/choriogonadotropin receptor (*LHCGR*), growth differentiation factor-9 (*GDF9*), anti-Müllerian hormone (*AMH*), and AMH type II receptor (*AMHR2*) genes [31–34]. However, the usefulness of sequence variants in clinical practice is limited due to inconclusive results from population-based studies [31,35]. Here, we identified several variants in genes such as *ESR1*, *ESR2*, *FSHB*, *FSHR*, *GDF9*, *LHCGR*, and *PRLR* correlating with the number of MII oocytes retrieved after ovarian stimulation; adding these variants to our model improved its predictive potential. Important variants were mainly found in genes encoding hormone receptors, for which the association with response to gonadotropins or oocyte maturation has already been confirmed in animal models. For example, mutations found in the LHCGR-encoding gene correlate with the empty follicle syndrome resulting in no oocytes retrieved during IVF [36]. Estrogen receptors—Erα and Erβ (encoded by *ESR1* and *ESR2* genes, respectively), are required for controlling oocyte meiotic resumption [37]. Mice lacking either FSHR or LHCGR gonadotropin receptors show impaired ovarian follicle growth and antrum formation and fail to develop preovulatory follicles (reviewed in [38]). The benefit of prolactin on bovine oocyte developmental capacity was shown to be mediated by cumulus cells containing prolactin receptors [39].

Simultaneous analysis of clinical data and sequence variants in a machine learning model may be weakened by the overlapping contribution of information from genetic and clinical data since the latter is derived from the former; at the same time, clinical data shows a stronger correlation with the observed phenotype. Indeed, the sequence variants directly correlating with the number of MII oocytes did not increase the model’s effectiveness. Rather than creating a model based on single variants, our approach focused on combinations of variants that were implemented in the model as genetic features and improved the prediction metrics compared to previous studies [40]. Interestingly, the average effect of genetic features on the prediction is higher than the effect of IVF protocol choice or PCOS presence. Genetic status is even more advantageous for the IV8-6 genetic feature, in comparison to stimulation data available from previous IVF cycles. Unlike the singly considered sequence variants, the genetic features showed a high occurrence in the population—haplotypes IV22-2 and IV41-8 were found in 58% and 65% of patients, respectively, and an average of 2.6 variants were found within the IV8-6 feature. As a result, many patients could benefit from the application of our clinical-genetic model.

The strength of our study lies in the number of methods used to select sequence variants associated with the number of MII oocytes and the use of multi-variant genetic features instead of single variants for modeling which increased the predictive potential of genetic data. In contrast to others [10,40], our approach was also strengthened by including data on retrospective stimulations. However, our study is limited by the lack of data on expression levels or structure-function predictions for variant proteins. A prospective clinical study on a large group of participants will also be needed to determine the concordance between our model’s predictions and actual stimulation results and verify the performance of our model in real-life.

In conclusion, genetic features increase the accuracy of predicting the number of MII oocytes retrieved after ovarian stimulation. The encouragement of a clinical-genetic model utilization will be realized by, firstly, incorporation in existing genetic test packages in flat-rate treatment plans without increasing prices, and secondly, by propagation to patients of the information of substantive add-on value enriching the standards of clinical procedures. When applied to clinical practice, we believe our model will improve personalized counseling and facilitate decision-making regarding the setting of gonadotropin doses to improve the safety and efficiency of stimulation protocols for IVF.

## Supporting information

S1 Appendix Supplementary protocols for next-generation sequencing. (PDF)

S2 Appendix Mathematical definitions, formulas and statistical hypotheses. (PDF)

S1 Table Detailed clinical characteristics of women in Group 1. (PDF)

S2 Table Characteristics of the 14 genes analyzed in this study, along with the number of sequence variants detected by genotyping. (PDF)

S3 Table Sequence variants in 14 reproduction-related genes identified by genotyping. (XLSX)

S4 Table Sequence variants with a positive effect on the number of MII oocytes identified by statistical tests. (PDF)

S5 Table Sequence variants with a negative effect on the number of MII oocytes identified by statistical tests. (PDF)

S6 Table Ranking of sequence variants sorted by their importance in predicting the number of MII oocytes. (PDF)

S7 Table Haplotypes with the greatest impact on the prediction of the number of MII oocytes. (PDF)

S8 Table Effect of genetic features on the predictive clinical-genetic model. (PDF)

S1 Fig Identification of sequence variants characteristic of MII groups by correspondence analysis (CA). The x- and y-axes show vertical and horizontal dimensions, respectively. Sequence variants on the chart are described with the gene symbol, variant name, and type of nucleotide change. Correspondence analysis allows the visualization of multidimensional datasets. In this study, it was used to present both patient groups and sequence variants on the same figure. The location of dots denoting the groups and variants shows dependencies in the dataset. The more the sequence variant is characteristic of a given MII group, the closer it is located on the visualization. The dependencies between variants and patient groups are stronger with increased distance from the origin of the coordinate system. Inertia covered I ∈ [0,1] shows the goodness of the model’s representation of the dataset, where 1 is the perfect representation. Here, the first two dimensions explain 83% of the inertia (x—0.63, y—0.20). Sequence variants *LHCGR* rs11887058 (NC_000002.12:g.48729336:C>T), *PRLR* rs112461 (NC_000005.10:g.35063190:A>T), and *ESR1* rs2207396 (NC_000006.12:g.152061247:G>A) are located closest to the MII group >11.
(PDF)

S2 Fig Representation of self-organizing map (SOM) trained on sequence variant data. Each dot represents different nodes of the model. The color of the dots for the subplots shows the share of observations of a given group in the nodes. The best matching unit (BMU) is defined as the neuron with the shortest distance to an observation. IVF stimulations are represented by the sequence variants of a patient, so the network’s neurons represent groups of similar observations in terms of genetic data. BMU was assigned for each IVF stimulation. SOM was trained, and after 100000 iterations achieved quantization error (QE) = 1.6. The neuron with coordinates (2,1) was the BMU for 53% of observations characterized by the number of MII oocytes of 0–2. Additionally, 86% of observations were characterized by the number of MII oocytes lower than seven. The size of a dot corresponds to the number of observations that has the node as the best matching unit (BMU). The results can be analyzed to find group structures in data, outlying observations, or features characteristic of a given group of observations. As each neuron on the map can be interpreted as a different group of observations, SOM was used to detect the most frequent sequence variants in neurons, where any patient group is over-represented. Identified sequence variants include *GDF9* rs11739194 (NC_000005.10:g.132865538T>C), *ESR2* rs928554 (NC_000014.9:g.64227477:C>T), *ESR1* rs2077647 (NC_000006.12:g.151807942:T>C), *GDF9* rs17166294 (NC_000005.10:g.132866205:T>C), *FSHB* rs676349 (NC_000011.10:g.30234435:A>G), *ESR1* rs2207396 (NC_000006.12:g.152061247:G>A), *LHCGR* rs62137532 (NC_000002.12:g.48687476:C>G), *ESR1* rs2273206 (NC_000006.12:g.152061176:G>T), *LHCGR* rs11887058 (NC_000002.12:g.48729336:C>T), and *ESR1* rs2273207 (NC_000006.12:g.152061190:A>G).
(PDF)

S3 Fig Construction of haplotypes for predicting the number of MII oocytes. **(A)** Details of the haplotype architecture. The block structures of Haplotypes 1, 2, and 3 constructed in Haploview software using the 4 Gamete Rule are shown. Each haplotype is displayed in a block with connections from one block to the next with thicker lines corresponding to more frequent crossings than thinner lines. A value of multiallelic D’ is shown in the crossing areas; this represents the level of recombination between the two blocks. The values next to the haplotypes show how often the haplotype occurred in the population. **(B)** Reduction of the number of variants in haplotypes. For sequence variants, the uniform manifold approximation and projection (UMAP) algorithm was trained to cluster the observations and extract groups of similar patients. A separate dataset was created for each haplotype reduction, containing the sequence variants that comprised the haplotype. UMAP’s visual representation allows for an analysis of whether the selected subset of variants in Haplotypes 1, 2, and 3 is sufficient to distinguish separate groups of patients. Each dot on the plot represents a single observation. A K-means algorithm was used to determine groups in the UMAP representation of the study population. A label that corresponds to its K-means detected group is assigned for each point. For example, seven separable groups are distinguished in the dataset for Haplotype 2. A label is assigned for each point, which corresponds to its group. A decision tree was then trained to find a group label based on the variants. To distinguish all observations from Group 1, which is the most frequent group (538 occurrences), 8 variants that make a path from the root of the tree to the first leaf node have to be the Ref variants. In other words, of the 41 variants that constructed the initial haplotype, 8 variants are responsible for detecting the largest group of the observations–and each variant has to have a Ref value. The newly created haplotype, called the IV41-8 genetic feature, as opposed to previous genetic features, is binary: If any of the eight selected variants is Alt, then the value of the feature is set to 0. The reduction process was also run for Haplotypes 1 and 3, resulting in genetic features IV22-2 and IV16-3.
(PDF)

S4 Fig Effect of variants in IV8-6 feature on MII oocyte predictions with regards to anti-Müllerian hormone (AMH) level. Range [−1.6, 2.4] represents the standardized number of alternative alleles in six variants comprising the genetic feature IV8-6 revealed by SOM analysis.
(PDF)
