# Supplementary material for: Personalized prediction of the secondary oocytes number after ovarian stimulation: A machine learning model based on clinical and genetic data
Source: PLoS Comput Biol. 2023 Apr 27;19(4):e1011020. doi: 10.1371/journal.pcbi.1011020 (PMC10138216; doi:10.1371/journal.pcbi.1011020)
Supplement: S1 Appendix — (PDF) [file pcbi.1011020.s001.pdf]

## S1 Appendix. Supplementary protocols for next-generation sequencing.

### Amplicon library preparation for next-generation sequencing

Briefly, target regions were PCR amplified; target amplification reactions were combined after thermal cycling, and amplicons were partially digested with FuPa reagent (ThermoFisher Scientific, Waltham, MA, USA). The barcoded adapters were then ligated to the amplicons with Switch Solution and DNA Ligase, and the amplicons were purified with Agencourt AMPure XP reagent and DynaMag-96 Side Magnet magnetic rack (all purchased from ThermoFisher Scientific, Waltham, MA, USA). Libraries were normalized to 100 pM concentration using a Library Equalizer Kit (ThermoFisher Scientific, Waltham, MA, USA). Barcoded and normalized libraries were combined and diluted to 8 pM. Template preparation and enrichment were performed using the Ion OneTouch 2 System instruments, Ion PGM Hi-Q View OT2 Kit reagents, and Ion PGM Hi-Q View OT2 Kit – 400 (all from ThermoFisher Scientific, Waltham, MA, USA) and the thermal cycling program according to the manufacturer's protocol.

### Sequencing reactions

The sequencing reactions of the template Ion Sphere Particles (ISPs) were conducted using the Ion PGM – Personal Genome Machine (Life Technologies, Carlsbad, CA, USA) instrument and Ion PGM Hi-Q View Sequencing Kit (ThermoFisher Scientific, Waltham, MA, USA) reagents. The sequencing reactions were loaded into semiconductor-based Ion 318 chips (ThermoFisher Scientific, Waltham, MA, USA), according to the manufacturer's protocol. Briefly, the sequencing primer was annealed to the template, the sequencing polymerase was added to the reaction, and the Ion 318 chip was loaded using the Ion Chip Minifuge (ThermoFisher Scientific, Waltham, MA, USA). The sequencing program was continued for 850 flows.

### Signal processing base calling, alignment, and variant calling

Signal processing base calling, alignment, and variant calling were performed using Ion Torrent Suite Server software version 5.12.2 (ThermoFisher Scientific, Waltham, MA, USA). The data processing parameters are described below. The Pre-BaseCaller Arguments for calibration were: BaseCaller --trim-qual-cutoff 15 --barcode-filter-minreads 20. BaseCaller Arguments were: BaseCaller --trim-qual-cutoff 15 --barcode-filter-minreads 20 --phred-table-file /opt/ion/config/phredTable.318.B5.h5. Alignment was performed against human genome assembly GRCh38.p2.mask1 with an argument as follows: tmap mapall ... -J 25 --end-repair 15 --do-repeat-clip --context stage1 map4. Sequence variants from GRCh38 in targeted regions were identified with the Ion variantCaller plug-in using variant caller version 5.12-28 and the germline_low_stringency_pgm parameters setting.
