# Supplementary material for: Personalized prediction of the secondary oocytes number after ovarian stimulation: A machine learning model based on clinical and genetic data
Source: PLoS Comput Biol. 2023 Apr 27;19(4):e1011020. doi: 10.1371/journal.pcbi.1011020 (PMC10138216; doi:10.1371/journal.pcbi.1011020)
Supplement: S2 Appendix — (PDF) [file pcbi.1011020.s002.pdf]

S2 Appendix. Mathematical definitions, formulas and statistical hypotheses.

Error metrics used to validate predictive model performance:

$$RMSE = \sqrt{\sum_{i=i}^{n} \frac{(\hat{y}_i - y_i)^2}{n}} \qquad (1)$$

$$MAE = \frac{1}{n} \sum_{i=i}^{n} |\widehat{y}_i - y_i| \qquad (2)$$

$$MAPE = \frac{1}{n} \sum_{i=i}^{n} \frac{|\widehat{y}_i - y_i|}{y_i} \times 100\% \qquad (3)$$

where $\hat{y}_i$ is the predicted number of MII oocytes for observation, y_i is the actual number of MII oocytes retrieved, and n is the population size.

Quantization error (QE) metric used to validate SOM performance:

$$QE = \frac{1}{n} \sum_{i=1}^{n} d(x_i, m_{c_i}) \qquad (4)$$

where n is the population size, d is the distance metric, x_i is the i-th observation and m_{c_i} is the BMU for the i-th observation.

Statistical hypotheses: Two separate groups were created for each variant: Group X with patients having the reference allele and Group Y with patients having the alternative allele of a sequence variant. Let F(u) and G(u) be the cumulative distribution functions of the distributions underlying Groups X and Y, respectively.

Testing for sequence variants that have a positive effect on the number of MII oocytes retrieved was performed as follows:

Mann–Whitney U hypothesis:
H_0 – F(u) = G(u)
H_1 – F(u) < G(u)

Kolmogorov–Smirnov hypothesis:
H_0 – F(u) ≥ G(u)
H_1 – F(u) < G(u)

Testing for sequence variants having a negative effect on the number of MII oocytes was performed as follows:

Mann–Whitney U hypothesis:
H_0 – F(u) = G(u)
H_1 – F(u) > G(u)

Kolmogorov–Smirnov hypothesis:
H_0 – F(u) ≤ G(u)
H_1 – F(u) > G(u)
