# Supplementary material for: Personalized prediction of the secondary oocytes number after ovarian stimulation: A machine learning model based on clinical and genetic data
Source: PLoS Comput Biol. 2023 Apr 27;19(4):e1011020. doi: 10.1371/journal.pcbi.1011020 (PMC10138216; doi:10.1371/journal.pcbi.1011020)
Supplement: S1 Table — (PDF) [file pcbi.1011020.s003.pdf]

S1 Table. Detailed clinical characteristics of women in Group 1. This study group consists of 5,779 patients and 8,574 IVF processes. Based on expert knowledge of the IVF domain, gynecologists selected a series of features that could affect the number of MII oocytes retrieved after ovarian stimulation. The features used to develop a clinical model are summarized. AMH—anti-Müllerian hormone, BMI—body mass index, E2— estradiol, IU—international unit, LH—luteinizing hormone, PCOS—polycystic ovary syndrome, PRG—progesterone.

**Characteristic**	**Mean ± SD**	**Range**
Age (years)	34.51 ± 4.54	18–46
Weight (kg)	65.19 ± 13.24	40–165
Height (cm)	166.80 ± 6.01	142–194
BMI (kg/m²)	23.42 ± 4.62	14.70–64.48
AMH level (ng/mL) [a)]	2.98 ± 2.42	0.02–14.88
E2 level (pg/mL)	18.62 ± 18.50	5.00–100.00
LH level (mIU/mL)	6.23 ± 4.63	0.10–19.90
PRG level (ng/mL)	0.32 ± 0.42	0.03–4.85
No. of follicles with sizes 2–8 mm	14.56 ± 9.02	0–80
No. of follicles with sizes 3–8 mm	7.79 ± 8.65	0–73
No. of follicles with sizes >11 mm	0.14 ± 0.82	0–19
No. of follicles with sizes >14 mm	0.11 ± 0.64	0–16
No. of follicles with sizes >18 mm	0.06 ± 0.39	0–10
No. of follicles with sizes 16–22 mm	0.06 ± 0.46	0–16
No. of pick-ups in the last 4 months	0.23 ± 0.50	0–4
No. of cumulus-denuded oocytes in the previous pick-up	8.26 ± 5.26	0–36
No. of MII oocytes in the previous pick-up	6.17 ± 4.10	0–32
No. of cumulus-denuded oocytes per cumulus in the previous pick-up	0.66 ± 0.43	0–5.5
No. of MII oocytes per cumulus in the previous pick-up	0.50 ± 0.36	0–4.33
Daily gonadotropin dose (IU) used in the previous process on Days 1–3 [b)]	240.97 ± 67.26	75–1,275
Daily gonadotropin dose (IU) used in the previous process on Days 4–7	207.22 ± 56.00	75–375
**Characteristic**	**Number of women**	**Percentage of population**
Cause of infertility—idiopathic	442	5.16%
Cause of infertility—tubal	602	7.02%
Cause of infertility—PCOS	406	4.74%
Cause of infertility—male factor	2,087	24.34%
Cause of infertility—male genetic factor	292	3.41%
Cause of infertility—female genetic factor	268	3.13%
Cause of infertility—thyroid disorders	1,348	15.72%
Cause of infertility—other endocrine disorders	448	5.23%
Cause of infertility—immunologic disorders	347	4.05%

Cause of infertility—recurrent implantation failure	1,088	12.69%
Cause of infertility—low ovarian reserve	518	6.04%
Cause of infertility—primary ovarian insufficiency	284	3.31%
Cause of infertility—endometriosis	557	6.50%

a) observations with the AMH level above 15ng/mL were removed from the database;
b) observations with the cumulative gonadotropin dose in Days 1–3 exceeding 1250 IU were removed from the database.
