# Supplementary material for: Personalized prediction of the secondary oocytes number after ovarian stimulation: A machine learning model based on clinical and genetic data
Source: PLoS Comput Biol. 2023 Apr 27;19(4):e1011020. doi: 10.1371/journal.pcbi.1011020 (PMC10138216; doi:10.1371/journal.pcbi.1011020)
Supplement: S2 Table — (PDF) [file pcbi.1011020.s004.pdf]

**S2 Table.** Characteristics of the 14 genes analyzed in this study, along with the number of sequence variants detected by genotyping. Biological process: GO:0048608—reproductive structure development, GO:0009755—hormone-mediated signaling pathway, GO:0022602—ovulation cycle process. Molecular function: GO:0005102—signaling receptor binding, GO:0030545—signaling receptor regulator activity.

| Gene symbol [a)] | Gene name [b)] | GO term | Chromosomal location [a)] | Reference sequence and location [b)] | Variants detected [c)] |
|---|---|---|---|---|---|
| *AMH* | anti-Mullerian hormone | GO:0048608, GO:0022602, GO:0005102, GO:0030545 | 19p13.3 | NC_000019.10 (2249323..2252073) | 26 |
| *AMHR2* | anti-Mullerian hormone receptor type 2 | GO:0048608 | 12q13.13 | NC_000012.12 (53423855..53431672) | 17 |
| *AR* | androgen receptor | GO:0048608, GO:0009755, GO:0005102 | Xq12 | NC_000023.11 (67544021..67730619) | 53 |
| *BMP15* | bone morphogenetic protein 15 | GO:0048608, GO:0005102, GO:0030545 | Xp11.22 | NC_000023.11 (50910735..50916641) | 15 |
| *ESR1* | estrogen receptor 1 | GO:0048608, GO:0009755, GO:0022602 | 6q25.1-q25.2 | NC_000006.12 (151656672..152129619) | 80 |
| *ESR2* | estrogen receptor 2 | GO:0009755, GO:0005102, GO:0030545 | 14q23.2-q23.3 | NC_000014.9 (64226707..64338613, complement) | 75 |
| *FSHB* | follicle-stimulating hormone subunit beta | GO:0048608, GO:0022602, GO:0005102, GO:0030545 | 11p14.1 | NC_000011.10 (30231014..30235194) | 19 |
| *FSHR* | follicle-stimulating hormone receptor | GO:0048608, GO:0009755, GO:0022602 | 2p16.3 | NC_000002.12 (48962157..49154515, complement) | 34 |
| *GDF9* | growth differentiation factor 9 | GO:0005102, GO:0030545 | 5q31.1 | NC_000005.10 (132861185..132866651, complement) | 33 |
| *LHB* | luteinizing hormone subunit beta | GO:0048608, GO:0009755, GO:0005102, GO:0030545 | 19q13.33 | NC_000019.10 (49015980..49019498, complement) | 22 |
| *LHCGR* | luteinizing hormone/choriogonadotropin receptor | GO:0048608, GO:0009755, GO:0022602 | 2p16.3 | NC_000002.12 (48686774..48755724, complement) | 44 |
| *PRL* | prolactin | GO:0005102, GO:0030545 | 6p22.3 | NC_000006.12 (22287246..22302835, complement) | 14 |

| | | | | | |
|---|---|---|---|---|---|
| *PRLR* | prolactin receptor | GO:0048608, GO:0009755, GO:0005102 | 5p13.2 | NC_000005.10 (35048756..35230487, complement) | 103 |
| *SOX3* | SRY-box transcription factor 3 | | Xq27.1 | NC_000023.11 (140502985..140505069, complement) | 9 |

[a] according to the HUGO Gene Nomenclature Committee (HGNC) database (www.genenames.org, accessed: 2022-04-05).
[b] according to the National Center for Biotechnology Information (NCBI) Gene database; based on GRCh38 human genome assembly (https://www.ncbi.nlm.nih.gov/gene/, accessed: 2022-04-05).
[c] details of identified sequence variants are presented in S3 Table.
