# Supplementary material for: Personalized prediction of the secondary oocytes number after ovarian stimulation: A machine learning model based on clinical and genetic data
Source: PLoS Comput Biol. 2023 Apr 27;19(4):e1011020. doi: 10.1371/journal.pcbi.1011020 (PMC10138216; doi:10.1371/journal.pcbi.1011020)
Supplement: S4 Table — (PDF) [file pcbi.1011020.s006.pdf]

S4 Table. Sequence variants with a positive effect on the number of MII oocytes identified by statistical tests.

**Gene**	**Variant**	**Reference allele**	**Alternative allele**	**Mann–Whitney U statistic**	***p*-value**	**Kolmogorov–Smirnov statistic**	***p*-value**
AR	rs189146053	C	A	8,940.50	0.00	0.46	0.00
LHCGR	rs11887058	C	T	72,783.50	0.00	0.12	0.01
LHCGR	rs10176989	C	A	42,881.50	0.00	0.14	0.01
PRLR	rs9292578	C	A	9,066.00	0.00	0.29	0.03
PRLR	rs115037164	C	A	9,604.50	0.01	0.29	0.03
PRLR	rs9292579	G	A	9,604.50	0.01	0.29	0.03
PRLR	rs142165087	G	C	9,604.50	0.01	0.29	0.03
PRLR	rs28365902	G	A	14,720.50	0.01	0.23	0.02
