# Supplementary material for: Personalized prediction of the secondary oocytes number after ovarian stimulation: A machine learning model based on clinical and genetic data
Source: PLoS Comput Biol. 2023 Apr 27;19(4):e1011020. doi: 10.1371/journal.pcbi.1011020 (PMC10138216; doi:10.1371/journal.pcbi.1011020)
Supplement: S5 Table — (PDF) [file pcbi.1011020.s007.pdf]

S5 Table. Sequence variants with a negative effect on the number of MII oocytes identified by statistical tests.

Gene	Variant	Reference allele	Alternative allele	Mann–Whitney U statistic	*p*-value	Kolmogorov–Smirnov statistic	*p*-value
AMH	rs8112524	G	A	39,418.50	0.00	0.17	0.00
AR	rs1337076	T	G	24,089.50	0.00	0.20	0.00
AR	rs150546551	A	G	6,156.50	0.00	0.30	0.01
AR	rs5919413	T	C	15,678.50	0.00	0.18	0.02
ESR1	rs4986934	T	C	51,220.50	0.00	0.12	0.01
ESR1	rs2228480	G	A	45,861.50	0.00	0.12	0.02
ESR1	rs2747648	C	T	36,921.50	0.00	0.15	0.00
ESR1	rs9341077	T	C	13,812.50	0.02	0.19	0.03
ESR1	rs2813543	A	G	40,263.50	0.01	0.11	0.04
ESR2	rs1255997	T	C	19,798.50	0.00	0.23	0.00
ESR2	rs8018687	T	C	14,554.00	0.00	0.25	0.00
ESR2	rs17225885	T	G	11,075.00	0.00	0.26	0.00
FSHB	rs78946483	T	G	11,601.50	0.00	0.20	0.03
FSHR	rs2072487	C	T	37,402.00	0.00	0.14	0.00
LHCGR	rs6545063	C	G	24,976.50	0.00	0.23	0.00
PRLR	rs56251626	C	G	12,007.00	0.00	0.22	0.01
PRL	rs849884	T	C	35,635.00	0.01	0.14	0.01
FSHR	rs1394205	C	T	45,611.00	0.00	0.11	0.02
