# Supplementary material for: Personalized prediction of the secondary oocytes number after ovarian stimulation: A machine learning model based on clinical and genetic data
Source: PLoS Comput Biol. 2023 Apr 27;19(4):e1011020. doi: 10.1371/journal.pcbi.1011020 (PMC10138216; doi:10.1371/journal.pcbi.1011020)
Supplement: S6 Table — (PDF) [file pcbi.1011020.s008.pdf]

S6 Table. Ranking of sequence variants sorted by their importance in predicting the number of MII oocytes. Here, 1 denotes the most important variant for the prediction of the number of MII oocytes. The ranking was determined by the Boruta and Boruta-SHAP methods. SHAP—SHapley Additive exPlanations; AMH—anti-Müllerian hormone.

Gene	Final ranking	Variant	Reference allele	Alternative allele	Boruta-SHAP ranking	Boruta with AMH ranking	Boruta without AMH ranking
GDF9	1	rs11739194	T	C	2	8	3
FSHB	2	rs676349	A	G	4	11	6
GDF9	3	rs17166294	T	C	17	35	14
ESR1	4	rs2273206	G	T	38	6	29
PRLR	5	rs56251626	C	G	5	64	7
ESR1	6	rs2207396	G	A	20	43	17
LHCGR	7	rs11887058	C	T	19	44	18
PRL	8	rs7739889	G	A	11	64	10
PRLR	9	rs37364	T	G	41	16	32
FSHR	10	rs1394205	C	T	14	64	12
ESR2	11	rs928554	C	T	21	50	19
PRLR	12	rs73091143	T	A	32	33	25
ESR1	13	rs2273207	A	G	25	52	20
ESR1	14	rs2228480	G	A	96	1	1
FSHR	15	rs6165	C	T	44	22	33
LHCGR	16	rs62137532	C	G	24	64	21
BMP15	17	rs3810682	C	G	30	60	24
PRLR	18	rs112461	A	T	29	64	23
BMP15	19	rs3897937	A	G	37	4	84
ESR1	20	rs2077647	T	C	39	7	84
