# Supplementary material for: Personalized prediction of the secondary oocytes number after ovarian stimulation: A machine learning model based on clinical and genetic data
Source: PLoS Comput Biol. 2023 Apr 27;19(4):e1011020. doi: 10.1371/journal.pcbi.1011020 (PMC10138216; doi:10.1371/journal.pcbi.1011020)
Supplement: S7 Table — (PDF) [file pcbi.1011020.s009.pdf]

S7 Table. Haplotypes with the greatest impact on the prediction of the number of MII oocytes. dbSNP Reference SNP number according to the National Center for Biotechnology Information (NCBI) dbSNP database (https://www.ncbi.nlm.nih.gov/snp/, accessed: 2022-04-05) is shown. Variants described compared to reference sequences listed in S2 Table and allele type is defined as Ref or Alt for variants corresponding to or differing from the reference sequence, respectively. Allele and haplotype frequencies are calculated for Group 2 consisting of 264 women.

**Haplotype**	**Gene (Chromosome) Reference sequence**	**dbSNP ID** [a]	**Sequence variant**	**Allele/type** [b]	**Allele frequency (%)** [c]	**Haplotype frequency** [c]
Haplotype 1	*FSHR* (chr2) NC_000002.12	NA	g.48755846=	A/Ref	1%	56%
		rs760106062	g.48961885=	C/Ref	<1%	
		rs149343367	g.48962033=	C/Ref	<1%	
		rs80111020	g.48962060=	A/Ref	9%	
		rs370297583	g.48962210=	C/Ref	<1%	
		rs72825259	g.48962487=	A/Ref	9%	
		rs6166	g.48962782C>T	T/Alt	71%	
		rs757713421	g.48963138=	G/Ref	<1%	
		rs202162496	g.48963491=	C/Ref	<1%	
		rs1674333268	g.48963599=	C/Ref	<1%	
		NA	g.48963670=	A/Ref	<1%	
		NA	g.48963773=	G/Ref	<1%	
		rs1444566790	g.48963859=	C/Ref	<1%	
		NA	g.48968573=	G/Ref	<1%	
		rs377301289	g.48968886=	A/Ref	<1%	
		NA	g.48982928=	A/Ref	<1%	
		rs75552966	g.48982977=	G/Ref	<1%	
		NA	g.48988980=	A/Ref	<1%	
		rs111883853	g.48989016=	C/Ref	2%	
		rs182717950	g.48989135=	A/Ref	<1%	
		rs779456539	g.48990555=	A/Ref	1%	
		rs754335673	g.48990619=	A/Ref	<1%	
Haplotype 2	*PRLR* (chr5) NC_000005.10	rs173627	g.35060399=	C/Ref	1%	55%
		rs249521	g.35060411=	A/Ref	1%	
		rs392279	g.35060629=	T/Ref	1%	
		NA	g.35060923=	C/Ref	<1%	
		rs918370607	g.35061009=	A/Ref	<1%	
		rs576920157	g.35061154=	G/Ref	<1%	
		rs387032	g.35061629=	T/Ref	16%	
		rs916915584	g.35061761=	T/Ref	<1%	
		rs147879910	g.35061871=	A/Ref	<1%	
		NA	g.35061973=	C/Ref	<1%	
		NA	g.35062253=	A/Ref	<1%	
		rs401694	g.35062516=	C/Ref	23%	
		NA	g.35062627=	A/Ref	<1%	
		NA	g.35063008=	G/Ref	<1%	
		rs1010119	g.35063015=	T/Ref	9%	
		rs531094388	g.35063098=	T/Ref	<1%	
		rs112461	g.35063190=	A/Ref	24%	
		rs569332036	g.35063323=	G/Ref	<1%	
		rs185993146	g.35064072=	A/Ref	<1%	

		NA	g.35064118=	C/Ref	<1%	
		rs73091133	g.35064133=	A/Ref	9%	
		rs1057829	g.35064358=	G/Ref	6%	
		rs1057828	g.35064413=	C/Ref	6%	
		rs73091139	g.35064637=	C/Ref	7%	
		rs540951852	g.35064682=	A/Ref	<1%	
		rs56251626	g.35064922=	C/Ref	7%	
		rs904632267	g.35065026=	G/Ref	<1%	
		rs931915988	g.35065030=	T/Ref	1%	
		rs764216661	g.35065384=	G/Ref	<1%	
		rs56255573	g.35065529=	T/Ref	<1%	
		rs62355478	g.35065548=	C/Ref	1%	
		NA	g.35065924=	G/Ref	<1%	
		rs1176242279	g.35065955=	T/Ref	<1%	
		rs79469561	g.35068136=	C/Ref	9%	
		rs78373811	g.35068146=	G/Ref	9%	
		rs754974807	g.35068265=	G/Ref	<1%	
		rs78921428	g.35068741=	T/Ref	<1%	
		rs186609463	g.35069864=	G/Ref	2%	
		rs72734529	g.35069940=	G/Ref	1%	
		rs73091143	g.35069955=	T/Ref	1%	
		NA	g.35070101C>A	A/Alt	<1%	
Haplotype 3	*GDF9* (chr5) NC_000005.10	NA	g.132865408=	A/Ref	<1%	36%
		rs17166283	g.132865453=	G/Ref	<1%	
		rs17166287	g.132865691=	T/Ref	<1%	
		rs76546692	g.132866060=	G/Ref	<1%	
		rs75061517	g.132866082=	T/Ref	31%	
		rs17166294	g.132866205=	T/Ref	29%	
		rs565694855	g.132866215=	C/Ref	<1%	
		rs140063257	g.132866313=	T/Ref	<1%	
		rs1475178166	g.132866484=	G/Ref	<1%	
		NA	g.132866553=	G/Ref	<1%	
		NA	g.132866657=	A/Ref	<1%	
		rs30178	g.132866707T>C	C/Alt	92%	
		rs30177	g.132866719C>G	G/Alt	92%	
		rs181795818	g.132866743=	G/Ref	<1%	
		NA	g.132866746=	C/Ref	<1%	
		NA	g.132867052=	C/Ref	7%	

a— dbSNP Reference SNP number according to the National Center for Biotechnology Information (NCBI) dbSNP database (https://www.ncbi.nlm.nih.gov/snp/, accessed: 2022-04-05);

b—allele type is defined as Ref for reference sequence variants and Alt for alternative sequence variants;

c—allele and haplotype frequencies were calculated for Group 2 consisting of 264 women.
