# Supplementary material for: Personalized prediction of the secondary oocytes number after ovarian stimulation: A machine learning model based on clinical and genetic data
Source: PLoS Comput Biol. 2023 Apr 27;19(4):e1011020. doi: 10.1371/journal.pcbi.1011020 (PMC10138216; doi:10.1371/journal.pcbi.1011020)
Supplement: S8 Table — (PDF) [file pcbi.1011020.s010.pdf]

S8 Table. Effect of genetic features on the predictive clinical-genetic model. In an iterative process, a total of 127 combinations of genetic features IV-CA, IV8-6, IV22-2, IV41-8, and IV16-3 were used in addition to clinical features to validate the effect on the predictive model. The reduction of the RMSE metric was calculated in each iteration with the RMSE metric of the clinical model equal to 3.53 oocytes used as a benchmark value.

**Added genetic features**	**RMSE reduction**
IV8-6, IV41-8, IV22-2	−0.18
IV8-6, IV41-8, IV16-3, IV22-2	−0.17
IV8-6, IV-CA, IV41-8, IV22-2	−0.17
IV8-6, IV41-8, IV16-3	−0.17
IV8-6, IV41-8	−0.17
IV8-6, IV-CA, IV41-8, IV16-3, IV22-2	−0.16
IV8-6, IV-CA, IV41-8	−0.16
IV8-6, IV16-3	−0.15
IV8-6, IV-CA, IV41-8, IV16-3	−0.15
IV8-6, IV22-2	−0.14
