# Supplementary material for: Personalized prediction of the secondary oocytes number after ovarian stimulation: A machine learning model based on clinical and genetic data
Source: PLoS Comput Biol. 2023 Apr 27;19(4):e1011020. doi: 10.1371/journal.pcbi.1011020 (PMC10138216; doi:10.1371/journal.pcbi.1011020)
Supplement: S1 Fig — The x- and y-axes show vertical and horizontal dimensions, respectively. Sequence variants on the chart are described with the gene symbol, variant name, and type of nucleotide change. Correspondence analysis allows the visualization of multidimensional datasets. In this study, it was used to present both patient groups and sequence variants on the same figure. The location of dots denoting the groups and variants shows dependencies in the dataset. The more the sequence variant is characteristic of a given MII group, the closer it is located on the visualization. The dependencies between variants and patient groups are stronger with increased distance from the origin of the coordinate system. Inertia covered I ∈ [0,1] shows the goodness of the model’s representation of the dataset, where 1 is the perfect representation. Here, the first two dimensions explain 83% of the inertia (x—0.63, y—0.20). Sequence variants LHCGR rs11887058 (NC_000002.12:g.48729336:C>T), PRLR rs112461 (NC_000005.10:g.35063190:A>T), and ESR1 rs2207396 (NC_000006.12:g.152061247:G>A) are located closest to the MII group >11. (PDF) [file pcbi.1011020.s011.pdf]

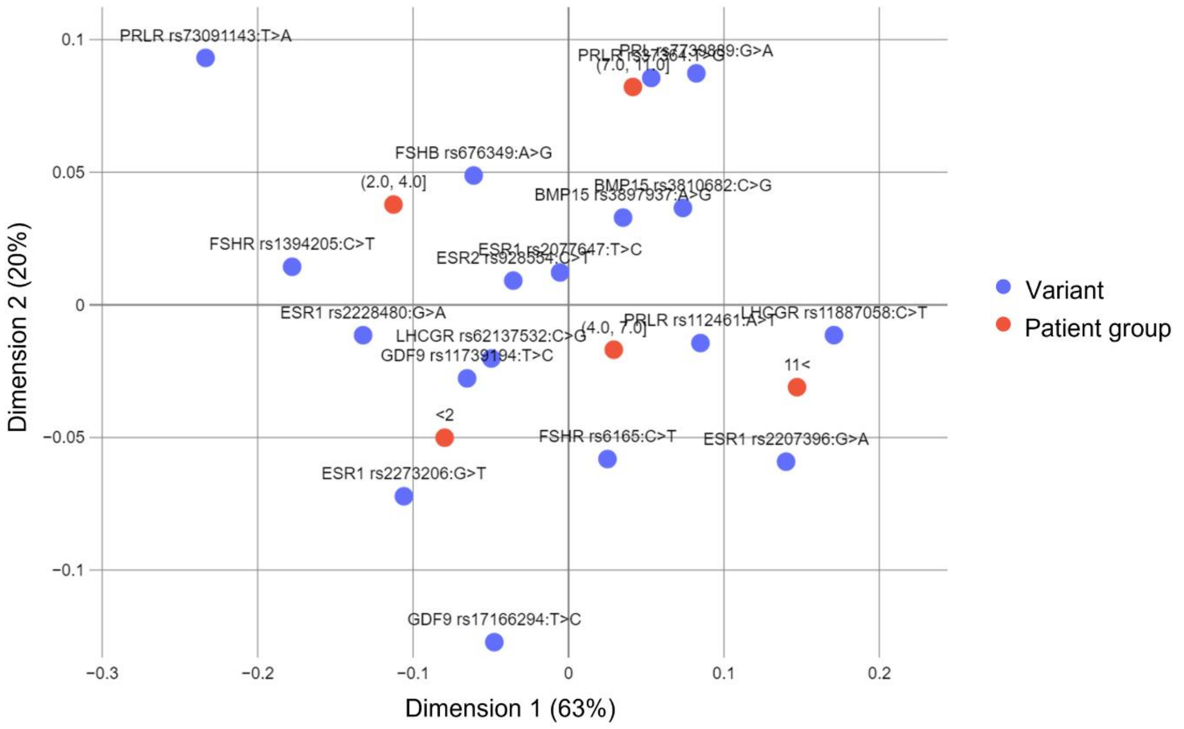

S1 Fig. Identification of sequence variants characteristic of MII groups by correspondence analysis (CA). The x- and y-axes show vertical and horizontal dimensions, respectively. Sequence variants on the chart are described with the gene symbol, variant name, and type of nucleotide change. Correspondence analysis allows the visualization of multidimensional datasets. In this study, it was used to present both patient groups and sequence variants on the same figure. The location of dots denoting the groups and variants shows dependencies in the dataset. The more the sequence variant is characteristic of a given MII group, the closer it is located on the visualization. The dependencies between variants and patient groups are stronger with increased distance from the origin of the coordinate system. Inertia covered $I \in [0,1]$ shows the goodness of the model's representation of the dataset, where 1 is the perfect representation. Here, the first two dimensions explain 83% of the inertia (x—0.63, y—0.20). Sequence variants *LHCGR* rs11887058 (NC_000002.12:g.48729336:C>T), *PRLR* rs112461 (NC_000005.10:g.35063190:A>T), and *ESR1* rs2207396 (NC_000006.12:g.152061247:G>A) are located closest to the MII group >11.
