# Supplementary material for: Personalized prediction of the secondary oocytes number after ovarian stimulation: A machine learning model based on clinical and genetic data
Source: PLoS Comput Biol. 2023 Apr 27;19(4):e1011020. doi: 10.1371/journal.pcbi.1011020 (PMC10138216; doi:10.1371/journal.pcbi.1011020)
Supplement: S2 Fig — Each dot represents different nodes of the model. The color of the dots for the subplots shows the share of observations of a given group in the nodes. The best matching unit (BMU) is defined as the neuron with the shortest distance to an observation. IVF stimulations are represented by the sequence variants of a patient, so the network’s neurons represent groups of similar observations in terms of genetic data. BMU was assigned for each IVF stimulation. SOM was trained, and after 100000 iterations achieved quantization error (QE) = 1.6. The neuron with coordinates (2,1) was the BMU for 53% of observations characterized by the number of MII oocytes of 0–2. Additionally, 86% of observations were characterized by the number of MII oocytes lower than seven. The size of a dot corresponds to the number of observations that has the node as the best matching unit (BMU). The results can be analyzed to find group structures in data, outlying observations, or features characteristic of a given group of observations. As each neuron on the map can be interpreted as a different group of observations, SOM was used to detect the most frequent sequence variants in neurons, where any patient group is over-represented. Identified sequence variants include GDF9 rs11739194 (NC_000005.10:g.132865538T>C), ESR2 rs928554 (NC_000014.9:g.64227477:C>T), ESR1 rs2077647 (NC_000006.12:g.151807942:T>C), GDF9 rs17166294 (NC_000005.10:g.132866205:T>C), FSHB rs676349 (NC_000011.10:g.30234435:A>G), ESR1 rs2207396 (NC_000006.12:g.152061247:G>A), LHCGR rs62137532 (NC_000002.12:g.48687476:C>G), ESR1 rs2273206 (NC_000006.12:g.152061176:G>T), LHCGR rs11887058 (NC_000002.12:g.48729336:C>T), and ESR1 rs2273207 (NC_000006.12:g.152061190:A>G). (PDF) [file pcbi.1011020.s012.pdf]

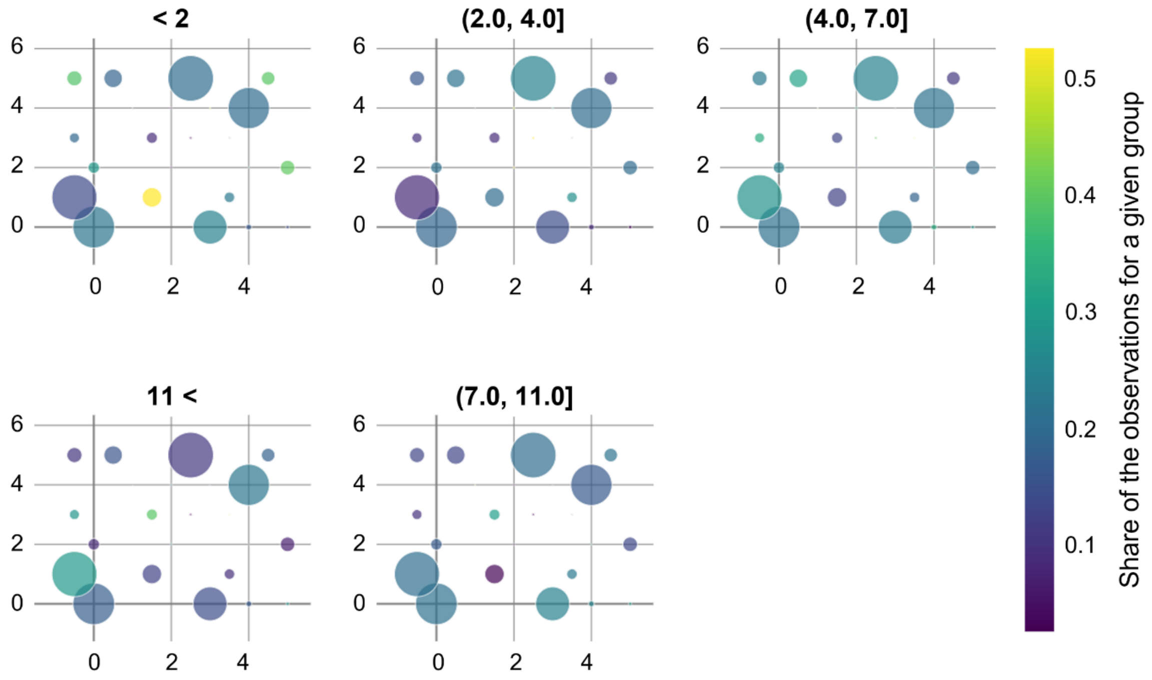

S2 Fig. Representation of self-organizing map (SOM) trained on sequence variant data. Each dot represents different nodes of the model. The color of the dots for the subplots shows the share of observations of a given group in the nodes. The best matching unit (BMU) is defined as the neuron with the shortest distance to an observation. IVF stimulations are represented by the sequence variants of a patient, so the network's neurons represent groups of similar observations in terms of genetic data. BMU was assigned for each IVF stimulation. SOM was trained, and after 100000 iterations achieved quantization error (QE) = 1.6. The neuron with coordinates (2,1) was the BMU for 53% of observations characterized by the number of MII oocytes of 0–2. Additionally, 86% of observations were characterized by the number of MII oocytes lower than seven. The size of a dot corresponds to the number of observations that has the node as the best matching unit (BMU). The results can be analyzed to find group structures in data, outlying observations, or features characteristic of a given group of observations. As each neuron on the map can be interpreted as a different group of observations, SOM was used to detect the most frequent sequence variants in neurons, where any patient group is over-represented. Identified sequence variants include *GDF9* rs11739194 (NC_000005.10:g.132865538T>C), *ESR2* rs928554 (NC_000014.9:g.64227477:C>T), *ESR1* rs2077647 (NC_000006.12:g.151807942:T>C), *GDF9* rs17166294 (NC_000005.10:g.132866205:T>C), *FSHB* rs676349 (NC_000011.10:g.30234435:A>G), *ESR1* rs2207396 (NC_000006.12:g.152061247:G>A), *LHCGR* rs62137532 (NC_000002.12:g.48687476:C>G), *ESR1* rs2273206 (NC_000006.12:g.152061176:G>T), *LHCGR* rs11887058 (NC_000002.12:g.48729336:C>T), and *ESR1* rs2273207 (NC_000006.12:g.152061190:A>G).
