# Supplementary material for: Personalized prediction of the secondary oocytes number after ovarian stimulation: A machine learning model based on clinical and genetic data
Source: PLoS Comput Biol. 2023 Apr 27;19(4):e1011020. doi: 10.1371/journal.pcbi.1011020 (PMC10138216; doi:10.1371/journal.pcbi.1011020)
Supplement: S3 Fig — (A) Details of the haplotype architecture. The block structures of Haplotypes 1, 2, and 3 constructed in Haploview software using the 4 Gamete Rule are shown. Each haplotype is displayed in a block with connections from one block to the next with thicker lines corresponding to more frequent crossings than thinner lines. A value of multiallelic D’ is shown in the crossing areas; this represents the level of recombination between the two blocks. The values next to the haplotypes show how often the haplotype occurred in the population. (B) Reduction of the number of variants in haplotypes. For sequence variants, the uniform manifold approximation and projection (UMAP) algorithm was trained to cluster the observations and extract groups of similar patients. A separate dataset was created for each haplotype reduction, containing the sequence variants that comprised the haplotype. UMAP’s visual representation allows for an analysis of whether the selected subset of variants in Haplotypes 1, 2, and 3 is sufficient to distinguish separate groups of patients. Each dot on the plot represents a single observation. A K-means algorithm was used to determine groups in the UMAP representation of the study population. A label that corresponds to its K-means detected group is assigned for each point. For example, seven separable groups are distinguished in the dataset for Haplotype 2. A label is assigned for each point, which corresponds to its group. A decision tree was then trained to find a group label based on the variants. To distinguish all observations from Group 1, which is the most frequent group (538 occurrences), 8 variants that make a path from the root of the tree to the first leaf node have to be the Ref variants. In other words, of the 41 variants that constructed the initial haplotype, 8 variants are responsible for detecting the largest group of the observations–and each variant has to have a Ref value. The newly created haplotype, called the IV41-8 genetic feature, [file pcbi.1011020.s013.pdf]

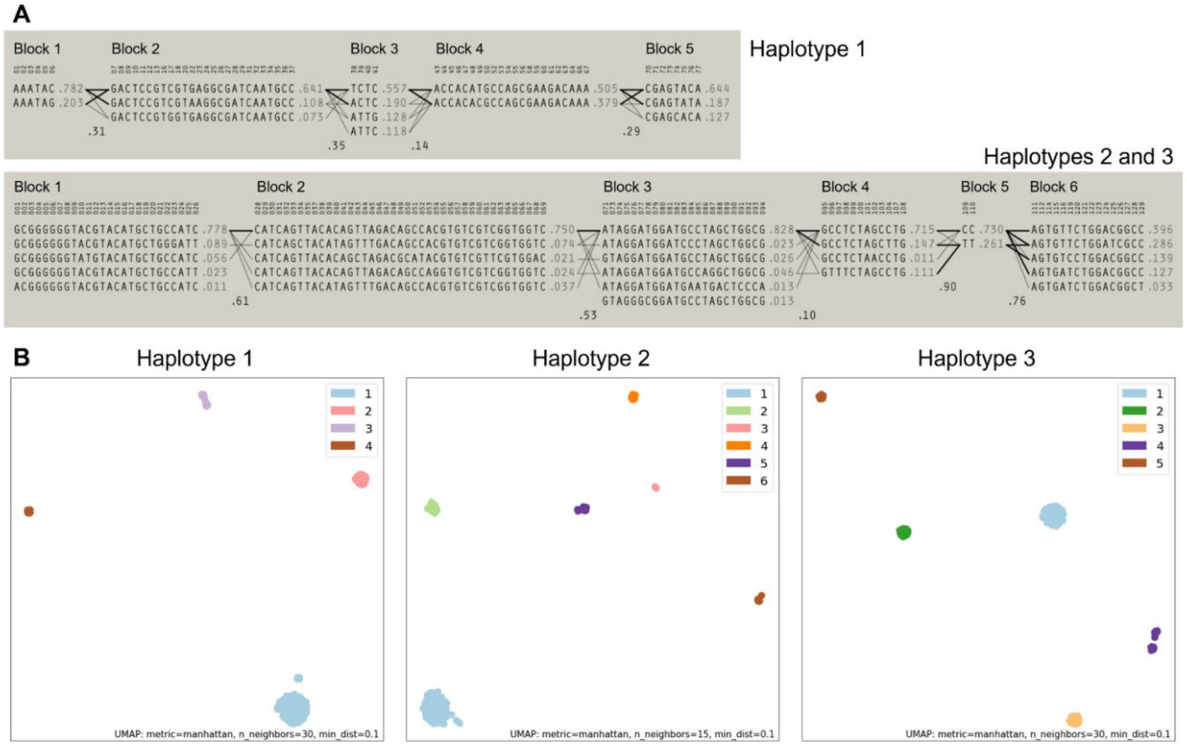

S3 Fig. Construction of haplotypes for predicting the number of MII oocytes. (A) Details of the haplotype architecture. The block structures of Haplotypes 1, 2, and 3 constructed in Haploview software using the 4 Gamete Rule are shown. Each haplotype is displayed in a block with connections from one block to the next with thicker lines corresponding to more frequent crossings than thinner lines. A value of multiallelic D' is shown in the crossing areas; this represents the level of recombination between the two blocks. The values next to the haplotypes show how often the haplotype occurred in the population. **(B)** Reduction of the number of variants in haplotypes. For sequence variants, the uniform manifold approximation and projection (UMAP) algorithm was trained to cluster the observations and extract groups of similar patients. A separate dataset was created for each haplotype reduction, containing the sequence variants that comprised the haplotype. UMAP’s visual representation allows for an analysis of whether the selected subset of variants in Haplotypes 1, 2, and 3 is sufficient to distinguish separate groups of patients. Each dot on the plot represents a single observation. A K-means algorithm was used to determine groups in the UMAP representation of the study population. A label that corresponds to its K-means detected group is assigned for each point. For example, seven separable groups are distinguished in the dataset for Haplotype 2. A label is assigned for each point, which corresponds to its group. A decision tree was then trained to find a group label based on the variants. To distinguish all observations from Group 1, which is the most frequent group (538 occurrences), 8 variants that make a path from the root of the tree to the first leaf node have to be the Ref variants. In other words, of the 41 variants that constructed the initial haplotype, 8 variants are responsible for detecting the largest group of the observations – and each variant has to have a Ref value. The newly created haplotype, called the IV41-8 genetic feature, as opposed to previous genetic features, is binary: If any of the eight selected variants is Alt, then the value of the feature is set to 0. The reduction process was also run for Haplotypes 1 and 3, resulting in genetic features IV22-2 and IV16-3.
