# Supplementary material for: Personalized prediction of the secondary oocytes number after ovarian stimulation: A machine learning model based on clinical and genetic data
Source: PLoS Comput Biol. 2023 Apr 27;19(4):e1011020. doi: 10.1371/journal.pcbi.1011020 (PMC10138216; doi:10.1371/journal.pcbi.1011020)
Supplement: S4 Fig — Range [−1.6, 2.4] represents the standardized number of alternative alleles in six variants comprising the genetic feature IV8-6 revealed by SOM analysis. (PDF) [file pcbi.1011020.s014.pdf]

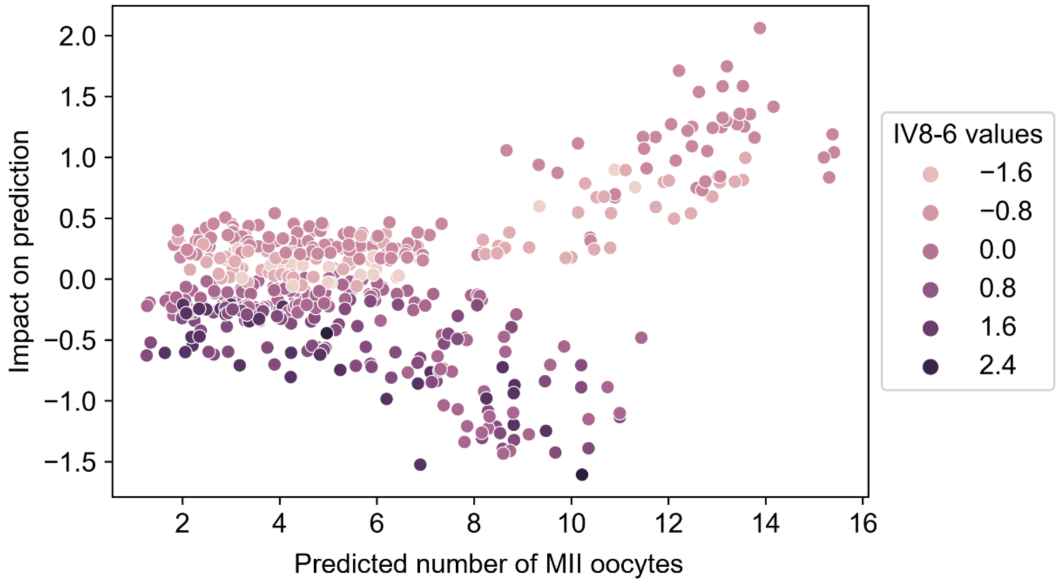

S4 Fig. Effect of variants in IV8-6 feature on MII oocyte predictions with regards to anti-Müllerian hormone (AMH) level. Range [−1.6, 2.4] represents the standardized number of alternative alleles in six variants comprising the genetic feature IV8-6 revealed by SOM analysis.
